# pVir and Bloody Diarrhea in *Campylobacter jejuni* Enteritis

**DOI:** 10.3201/eid1106.041052

**Published:** 2005-06

**Authors:** Dobryan M. Tracz, Monika Keelan, Jasmine Ahmed-Bentley, Amera Gibreel, Kinga Kowalewska-Grochowska, Diane E. Taylor

**Affiliations:** * University of Alberta, Edmonton, Alberta, Canada;; † Public Health Agency of Canada, Edmonton, Alberta, Canada;; ‡ University of Alberta Hospital Microbiology Laboratory and Provincial Laboratory for Public Health (Microbiology), Edmonton, Alberta, Canada

**Keywords:** antibiotic resistance, Campylobacter jejuni, virulence plasmid, pVir

## Abstract

The plasmid pVir may play a role in the virulence of *Campylobacter jejuni*, a leading cause of bacterial gastroenteritis. The pVir plasmid was identified in 17% of 104 *C. jejuni* clinical isolates studied and was significantly associated with the occurrence of blood in patient stool, a marker of invasive infection. The pVir plasmid was not associated with greater occurrence of diarrhea, fever, pain, vomiting, or need for patient hospitalization. Isolates containing pVir were also associated with the presence of a tetracycline-resistance plasmid, but pVir did not transfer with tetracycline-resistance plasmids to recipient strains of *C. jejuni*. The association of pVir and bloody stool suggests that pVir may be clinically relevant in *C. jejuni* infections.

*Campylobacter jejuni* is a major foodborne pathogen and a leading cause of bacterial gastroenteritis (1,2). Infection with *C. jejuni* can result in a wide array of clinical symptoms, including diarrhea, fever, abdominal pain, and vomiting, as well as bloody stool with severe invasive infection (3). Various virulence factors, which allow for adherence, colonization, and invasion of the intestinal epithelium, have been proposed to contribute to the pathogenesis of *C. jejuni* (4). Potential virulence components include flagella (5,6), invasion proteins (7), and toxins (8,9). Although the genome of *C. jejuni* has been sequenced (10), its mechanisms of pathogenicity remain poorly understood (11).

A number of bacterial enteric pathogens contain plasmids that contribute to pathogenesis, including *Shigella* sp (12), *Salmonella* sp (13), and enteropathogenic *Escherichia coli* (14). No evidence was seen for the involvement of plasmids in the virulence of *C. jejuni* until Bacon et al. (15) identified plasmid pVir in strain 81-176. pVir is an ≈37.5-kb plasmid that contains components of a type IV secretion system (T4SS) (15,16) known to be important for the virulence of a number of major bacterial pathogens (17). Bacon et al. (15) suggested that the pVir plasmid is important in vitro for both adherence and invasion of intestinal epithelial cells in culture. In a survey of fresh clinical isolates of *C. jejuni* from Thailand, 10% (n = 58) contained pVir (15).

*C. jejuni* gastroenteritis is primarily self-limiting and is usually treated by supportive therapy (fluid and electrolyte replacement) (3). Erythromycin is the drug of choice for treating severe clinical infections with *C. jejuni* (18), and fortunately the prevalence of erythromycin resistance has remained low (19). Worldwide, tetracycline resistance in *C. jejuni* is high: 56% in Canada (20) and up to 95% in Thailand (21). In Alberta, Canada, only 8.6% of human clinical isolates were tetracycline-resistant in 1981 (22). In *C. jejuni*, tetracycline resistance is primarily mediated by plasmids that carry the *tet*(*O*) gene (23,24). The Tet(O) protein binds to the bacterial ribosome and displaces tetracycline (25,26). The aim of this study was to investigate the prevalence of the pVir plasmid in *C. jejuni* isolated from clinical specimens in Alberta and the relationship of pVir to the clinical expression of the disease in gastroenteritis.

## Materials and Methods

### Source of *C. jejuni* Isolates

We obtained a random sample of 104 human isolates of *C. jejuni* (fresh and frozen at –70°C in double-strength skim milk) cultured from stool samples from 1999 to 2002 at the University of Alberta Hospital and Provincial Laboratory for Public Health (Microbiology) in Edmonton, Alberta, Canada, for this study. All stool specimens were routinely cultured for enteric pathogens tested according to standard laboratory protocols. Most *C. jejuni* isolates were from northern Alberta and represented ≈10% of *C. jejuni* infections in Alberta reported annually to Health Canada. The Health Research Ethics Board (Biomedical Panel) of the University of Alberta approved the protocol for access to human isolates and collection of patient information for this study.

### Collection of Clinical Data

Clinical data were collected by letter or phone from family physicians' offices and emergency medical records and included information on age, sex, place of residence, travel within the last month, coexisting conditions, symptoms (diarrhea, bloody diarrhea, pain, fever, vomiting) and their duration (interval from symptom onset to initial evaluation), hospitalization (if needed), antimicrobial therapy, and complications. Fever was defined as a complaint of fever stated by the patient or a documented temperature of >38°C. Blood in the stool was observed by the patient and reported to the attending physician. The investigators conducting the chart review and laboratory analyses (pVir screening, antimicrobial susceptibility testing) were blinded to each other's data.

### Culture of *C. jejuni* Isolates

Isolates of *C. jejuni* were spread on brain heart infusion agar (Difco-Becton-Dickinson, Sparks, MD, USA), supplemented with 0.4% yeast extract (Difco), and incubated at 37°C in microaerobic conditions (5% CO_2_, 10% H_2_, 85% N_2_) for 48 h. Isolates were not passaged more than once. All isolates were stored frozen in brain heart infusion broth (Difco) with 20% glycerol at –80°C.

### Antimicrobial Susceptibility and Plasmid Isolation

*C. jejuni* susceptibility was tested by using the disk diffusion method; the following antimicrobial disks (Oxoid, Nepean, Ontario, Canada) were included: tetracycline (30 μg), kanamycin (30 μg), erythromycin (15 μg), chloramphenicol (30 μg), and nalidixic acid (30 μg). All nalidixic acid-resistant isolates were further tested for resistance to ciprofloxacin (1 μg). Susceptibility testing to all antimicrobial agents was carried out on Mueller-Hinton agar plates that were spread with a 0.5 McFarland standard suspension of *C. jejuni* in phosphate-buffered saline (Sigma, St. Louis, MO, USA) and incubated for 48 h at 37°C under microaerobic conditions. Zones of inhibition were measured as described by Gaudreau and Gilbert (27). Plasmid isolations were performed by using a Qiagen Midi or Mini Plasmid Kit (Qiagen, Mississauga, Ontario, Canada), by alkaline lysis (28) or by a method used for *Helicobacter pylori* (29). Tetracycline resistance detected by disk diffusion was confirmed by Etest (Oxoid) and conventional agar dilution.

### DNA-DNA Hybridizations for pVir

Purified plasmid DNA from *C. jejuni* clinical isolates was applied to nitrocellulose paper (Osmonics, Westborough, MA, USA) and air dried. Plasmid pMS11EH was used as a negative control, and plasmid DNA isolated from *C. jejuni* 81-176 was used as a positive control. Plasmid DNA was denatured in 0.5 mol/L NaOH, 0.15 mol/L NaCl for 5 min, neutralized twice in 10 mmol/L Tris-HCl, 0.15 mol/L NaCl, pH 7.5 for 5 min each time, soaked in 2× SSPE solution (20× SSPE consists of 3.0 mol/L NaCl, 0.2 mol/L NaH_2_PO_4_, and 0.02 mol/L EDTA) for 5 min, air dried, and baked overnight at 65°C.

The *cjp5* (*virB11*) DNA probe was prepared with primers *virB11* Fwd (5´ GAACAGGAAGTGGAAAAACTAGC 3´) and *virB11* Rev (5´ TTCCGCATTGGGCTATATG 3´) (15) that were used to amplify by polymerase chain reaction (PCR) a 708-bp product from within the *cjp5* gene on pVir. Conditions for the PCR were as follows: initial denaturation at 95°C for 1 min, followed by 30 cycles of denaturation (1 min, 95°C), annealing of primers (1 min, 50°C) and primer extension (1 min, 72°C). PCR was performed in a BioRad Gene Cycler (BioRad, Mississauga, Ontario, Canada). The *cjp5* PCR product was purified with a PCR purification kit (Qiagen) and eluted with TE (Tris-EDTA) buffer. DNA was denatured after boiling for 5 min, and the tube was then placed directly on ice. A random primers DNA labeling system (Gibco, Burlington, ON) was used to prepare the ^32^P-labeled *cjp5* DNA probe. The ^32^P-labeled *cjp5* DNA probe was used immediately in DNA-DNA hybridizations to screen isolated *C. jejuni* plasmids for the presence of pVir, under the same stringency conditions defined by Bacon and coworkers (15). Hybridizations were performed in a solution consisting of 6× SSC (20× consists of 3.0 mol/L NaCl and 0.3 mol/L sodium acetate), 5× Denhart solution (50× Denhardt solution consists of 1% wt/vol Ficoll 400 (Sigma), 1% wt/vol polyvinylpyrrolidone (Sigma), and 1% wt/vol bovine serum albumin (Sigma, Fraction V), 0.1% sodium dodecyl sulfate (SDS, BioRad) and 100 μg/mL herring sperm DNA (Invitrogen, Burlington, Ontario, Canada). The ^32^P-labeled *cjp5* DNA probe was added directly to the hybridization solution in the tube, mixed, and incubated overnight (18 h) at 50°C. The probed nitrocellulose paper was washed in 0.5× SSC 4 times, dried, and then exposed to BioMax MS Film (Eastman Kodak Company, Rochester, NY, USA) overnight at –70°C.

### Identifying Tetracycline-Resistance Plasmids

Tetracycline-resistance plasmids were identified by a PCR screen for the *tet*(*O*) gene on purified plasmid preparations. Primers *tet*(*O*) Fwd (5´ GGCGTTTTGTTTATGTGCG 3´) and *tet*(*O*) Rev (5´ ATGGACAACCCGACAGAAGC 3´) were used to amplify a 559-bp fragment of the *tet*(*O*) gene from isolated *C. jejuni* plasmid DNA (15,30). PCR conditions were the same as for the *cjp5* PCR described above. Gel electrophoresis was used to confirm the presence of an ≈40-kb plasmid.

### Transfer of Plasmids between *C. jejuni* Strains

The transfer of plasmids from *C. jejuni* clinical isolate 23-51 (containing both a tetracycline-resistance plasmid and pVir) to *C. jejuni* UA 543 (a nalidixic acid–resistant, tetracycline-susceptible recipient strain with no plasmids) was carried out (23). Suspensions of donor strain 23-51 and UA 543 recipient strains were mixed in ratios of 1:4 and 1:6, centrifuged, and resuspended in 100 μL of Lennox broth (Difco). Mating suspensions were inoculated onto a 0.22-μm Millipore filter (Millipore Corporation, Nepean, Ontario, Canada), placed on brain heart infusion agar, and incubated for 24 h. Cultures were resuspended in 1 mL of phosphate-buffered saline, diluted, and plated onto Mueller-Hinton agar containing 25 μg/mL tetracycline and 50 μg/mL of nalidixic acid. Plasmids were isolated from the transconjugants by a Qiagen Mini Kit. The *cjp5* DNA probe was used to screen for pVir as described above.

### Statistical Analysis

The Fisher exact test was used to test the significance (p<0.05) of the association between pVir and clinical symptoms, association of pVir and tetracycline-resistance plasmids, and the frequency of tetracycline resistance from this study and a previous study (22).

## Results

### Clinical Data

The patients were 7 months to 87 years of age (average 35 years); 47% were female. Ninety-five percent of the patients resided in Alberta, and the remaining 5% were from other Canadian provinces. Only 10 patients reported a history of travel outside Canada before becoming ill, namely to Mexico (3 patients), Alaska, Nicaragua, Ecuador, England, India, Lebanon, and Africa (1 patient each). The most commonly reported clinical symptom was diarrhea (99%); the exception was in an 86-year-old woman, who had an ileus. Other commonly reported symptoms were abdominal pain (83%) and fever (77%). Bloody stool was reported in 27% of patients and vomiting in 30% of patients; 18% of patients were hospitalized for treatment of severe dehydration. Fifty-eight patients received antimicrobial therapy: 34 ciprofloxacin (1 was switched to erythromycin after culture results, 2 received ciprofloxacin in combination with metronidazole), 20 macrolides (13 erythromycin, 5 clarithromycin, 3 azithromycin), 2 metronidazole (1 in combination with cephalexin), 1 amoxicillin, and 1 cotrimoxazole. Sixteen patients had other associated conditions: 4 cardiac disorders (2 arrhythmias, 2 ischemic heart disease), 3 hypertension (1 also had chronic obstructive pulmonary disease), 3 diabetes, 2 neoplasms (1 leukemia, 1 Wilm tumor), 2 gastrointestinal disorders (1 ulcerative colitis, 1 irritable bowel), 1 hepatitis C, and 1 seizure. Campylobacteremia with febrile seizures developed in a previously healthy patient.

### Antimicrobial Susceptibility

The antimicrobial susceptibility of 104 human isolates of *C. jejuni* is shown in Table 1. Tetracycline resistance was identified in 63 isolates (60%); of these, 4 isolates were also resistant to nalidixic acid, 2 isolates were also resistant to kanamycin, and 1 isolate was also resistant to both nalidixic acid and kanamycin. Three of the 4 nalidixic acid–resistant isolates were also resistant to ciprofloxacin. In the tetracycline-sensitive isolates, no resistance to other antimicrobial agents was detected.

**Table 1:** Antimicrobial resistance frequencies in human clinical isolates of *Campylobacter jejuni* (n = 104) as determined by antimicrobial disk diffusion, 1999–2002

Antimicrobial agent	Resistance frequency (%)
Tetracycline	60
Nalidixic acid	4
Ciprofloxacin	3
Kanamycin	3
Erythromycin	0
Chloramphenicol	0

### Plasmid Content

Tetracycline-resistance plasmids were found in 50 (79%) of 63 tetracycline-resistant isolates. DNA-DNA hybridizations for the *cjp5* gene on pVir determined that 18 (17%) of 104 *C. jejuni* isolates contained the pVir plasmid. Stocked frozen clinical isolates had a slightly higher frequency of pVir than fresh clinical isolates (18% vs. 13%, respectively), most likely because of sampling error. Tetracycline-resistance plasmids were found in 17 (94%) of 18 pVir-positive *C. jejuni* isolates compared with 33 (38%) of 86 pVir-negative isolates. The presence of pVir plasmids was associated with the presence of tetracycline-resistance plasmids (p<0.00001). Alternatively, 33 (66%) of the 50 isolates that contained tetracycline-resistance plasmids did not contain pVir, demonstrating that plasmid-mediated tetracycline resistance does not occur exclusively with pVir.

### Relationship of Signs and Symptoms to Plasmid Content

Pain, diarrhea, vomiting, and fever were equally likely to occur in all *C. jejuni* infections, regardless of the presence of pVir (Table 2). However, 53% of patients infected with pVir-positive *C. jejuni* strains had bloody stool, as opposed to 21% of patients infected with pVir-negative *C. jejuni* strains (p = 0.011). pVir was not associated with age, sex, antimicrobial therapy, coexisting conditions, or travel. The patient with campylobacteremia and febrile seizures was infected with a pVir-negative *C. jejuni*. None of the patients infected with pVir-positive *C. jejuni* strains had a history of travel outside of Alberta in the week preceding the illness, and all of the patients who traveled outside of Alberta were infected with pVir-negative *C. jejuni* strains.

**Table 2:** Association of pVir plasmid with clinical symptoms in patients with *Campylobacter jejuni* gastroenteritis, Alberta, Canada, 1999–2002

pVir	Clinical symptom (%)						
	Pain	Diarrhea	Vomiting	Blood in stool	Fever	Hospitalization	Duration <7 days (%)
Present (n = 18)*	83 (n = 18)	100 (n = 18)	28 (n = 15)	53† (n = 17)	59 (n = 17)	22 (n = 18)	77 (n = 14)
Absent (n = 86)	80 (n = 72)	99 (n = 85)	30 (n = 73)	21 (n = 76)	64 (n = 64)	17 (n = 86)	82 (n = 68)

*Samples sizes (n) for each clinical symptom differ as symptoms were not known for each patient. †p<0.05, presence vs. absence of pVir.

### Transfer of Plasmids between *C. jejuni* Strains

Tetracycline-resistant transconjugants contained ≈40-kb plasmids that carried *tet*(*O*) but were negative for pVir in DNA-DNA hybridizations with the *cjp5* probe (data not shown). This finding confirmed the finding of Bacon et al. (15) that pVir could not be transferred with tetracycline-resistance plasmids in conjugal mating between *C. jejuni* strains.

## Discussion

The role of pVir in human *C. jejuni* infections has not been investigated previously. Bacon et al. (15,16) presented evidence for the role of pVir in the virulence of *C. jejuni* based on in vitro cell-culture experiments and limited animal data with the ferret model (15). In our study, symptoms associated with *C. jejuni* infection were correlated with the presence or absence of pVir plasmids. Symptoms varied from mild discomfort to severe cases with bloody diarrhea. Travel and coexisting medical conditions were not predictors of severity of *C. jejuni* infections and were not associated with a higher frequency of blood in the stool or pVir-positive *C. jejuni* infections. Although the pVir virulence plasmid did not correlate with most clinical symptoms, patients infected with a pVir-positive *C. jejuni* strain were more likely to produce bloody stool than those infected with a pVir-negative strain. The time course of the disease may have affected whether or not blood was observed in the stool sample. The clinical data collected reflect both the observations of the patient as reported to the physician and the physician's records. Accordingly, these conclusions are based upon the necessary limitations of this retrospective study of patients from different unconnected study sites within a region, with perhaps some differences in diagnostic definitions.

Bloody stool in *C. jejuni* gastroenteritis indicates the progression of the infection into the tissues of the colon and rectum (3). This invasion of the intestinal epithelium is responsible for the mucosal damage and inflammatory lesions seen in *C. jejuni* infections and is a major component of pathogenesis, although the mechanism is currently unknown (11). pVir was previously found to be important for the in vitro invasion of intestinal epithelial cell lines (15,16). The association of pVir in *C. jejuni* with bloody diarrhea in a clinical setting supports the role of pVir for in vivo epithelial cell invasion and stresses its potential as a marker for the risk of developing a more severe clinical infection.

The lack of association of pVir with bloody stool in a small proportion of patients suggests that other virulence determinants are likely to be involved in severe *C. jejuni* infections, as are several other host factors that determine the clinical expression of the disease (3). Further studies are necessary to clarify these aspects of clinical *C. jejuni* infection.

Large variations in invasion frequency have been observed among strains of *C. jejuni* (4). Fearnely et al. (31) found that *C. jejuni* strains can either invade cell cultures at high frequencies (hyperinvasive) or have low invasive potential. Subsets of the *C. jejuni* strains may exist that use different mechanisms to produce disease (15,32). Whether or not the pVir virulence plasmid is the defining feature of a hyperinvasive subset of *C. jejuni* strains remains to be determined.

The finding that the pVir virulence plasmid is present in *C. jejuni* isolates containing a tetracycline-resistance plasmid is of considerable interest. One potential explanation for this association is the impact on the invasive ability of the strain. Variation in the invasion frequencies of *C. jejuni* 81-176 pVir gene mutants has been observed (33) and may be due to the functional redundancy of T4SS genes found on pVir and a tetracycline-resistance plasmid (16). This finding may, in part, explain the dependence of pVir on the tetracycline-resistance plasmid observed in this study.

The prevalence of tetracycline resistance in *C. jejuni* in our study (60%) represents a significant increase from 8.6% in 1981 (p<0.0001) (22), but the frequency of resistance to erythromycin or ciprofloxacin has remained low. These results confirm those of other Canadian studies that have identified increasing levels of tetracycline resistance and low levels of erythromycin resistance (20,34,35). Considering that erythromycin is the drug of choice for treating *C. jejuni* gastroenteritis, that its efficacy has not been compromised by the emergence of resistance is surprising. In our study, a low level of resistance to ciprofloxacin, commonly prescribed to prevent travelers' diarrhea, is also a surprising finding. A temporal link between veterinary use of fluoroquinolones and the emergence of fluoroquinolone resistance in human isolates of *C. jejuni* coincided with the licensing of fluoroquinolones, such as enrofloxacin, in the late 1980s and early 1990s in the Netherlands, Spain, United Kingdom, the United States, and Canada (36). In the eastern Canadian province of Quebec, resistance to ciprofloxacin increased from 13% in 1995 to 47% in 2001 (20,37). Approval for fluoroquinolone as a therapeutic agent for use in agriculture in Canada was withdrawn in 1997 (38) and may explain the low prevalence of ciprofloxacin-resistant *C. jejuni* isolated from human specimens in the western Canadian province of Alberta.

Serotyping was not conducted in our study since on its own it is not a useful discriminating marker for the ability of *C. jejuni* isolates to cause severe gastroenteritis. Molecular subtyping studies in the United States reported virtually identical isolates of *C. jejuni* in locally purchased retail poultry products and human infections (39). Future studies will continue to investigate the association of pVir-positive *C. jejuni* infections with the presence of blood in stool and employ a variety of genotypic tools (molecular typing, DNA microarrays) and phenotypic assays (i.e., invasion) to identify the characteristics of *C. jejuni* isolates from animal and human sources that allow them to be effective pathogens in humans.
